# Challenges in treatment of renal echinococcosis with gross hydatiduria and unsalvageable kidney: a case report

**DOI:** 10.1186/s13256-021-02992-2

**Published:** 2021-09-29

**Authors:** Sameera Shuaibi, Abdelrahman AlAshqar, Munirah Alabdulhadi, Wasl Al-Adsani

**Affiliations:** 1 Department of Internal Medicine, Al-Amiri Hospital, Kuwait City, Kuwait; 2 Department of Obstetrics, Gynecology and Reproductive Sciences, Yale University School of Medicine, 333 Cedar Street, New Haven, CT 06510 USA; 3 Mubarak Al-Kabeer Hospital, Kuwait City, Kuwait; 4 Department of Internal Medicine, Mubarak Al-Kabeer Hospital, Kuwait City, Kuwait; 5 Infectious Diseases Hospital, Kuwait City, Kuwait

**Keywords:** Albendazole, Case report, Hydatid cyst, Hydatiduria, Renal echinococcosis

## Abstract

**Introduction:**

Renal echinococcosis is of rare occurrence, and although often asymptomatic, it can present with various mild to drastic presentations, of which hydatiduria is pathognomonic. Diagnosis can be preliminarily established by imaging, and treatment is primarily surgical. We present a patient with renal echinococcosis treated successfully with exclusive antiparasitic pharmacotherapy after refusing surgery despite extensive renal involvement. We hope through this report to help establish future solid guidelines regarding this uncommon therapeutic approach.

**Case presentation:**

This is a case of a 49-year-old Syrian shepherd presenting with flank pain and passage of grape-skin-like structures in urine. A diagnosis of renal echinococcosis with hydatiduria and significant parenchymal destruction was established based on exposure history, positive serology, imaging findings, and renal scintigraphy. After proper counseling, the patient refused nephrectomy and was therefore started on dual pharmacotherapy (albendazole and praziquantel) and is having an uneventful follow-up and a satisfactory response to treatment.

**Conclusion:**

This case embodies the daily challenges physicians navigate as they uphold the ethical principles of their practice and support their patients’ autonomy while delivering the best standards of care and consulting the scientific evidence. Although surgery is the cornerstone of renal echinococcosis treatment, treating physicians should be prepared to tackle situations where surgery cannot be done and offer the best next available option for patients who refuse surgery. As data on exclusive pharmacotherapy are limited, future research should thoroughly investigate the efficacy of this uncommon approach and outline reliable recommendations, facilitating future clinical decision-making in this avenue.

## Introduction

Human echinococcosis, commonly known as hydatid disease, is an underreported, yet a considerably morbid, zoonotic infection that has been classified by the World Health Organization (WHO) as one of the major neglected tropical diseases as published in their first report on these entities [1]. Although it can be asymptomatic for years, the chronic nature of echinococcosis and its potential to spread widely to many organs can lead to debilitating consequences and troublesome treatment regimens [1]. Whereas echinococcosis is endemic to certain regions, such as the Middle East, sub-Saharan Africa, and Central and South America, cases have been reported worldwide, [2] and data indicate that echinococcosis is reemerging as a major public health issue, mandating a heightened level of attention [3].

Echinococcosis has a predilection to affect most commonly the liver followed by the lungs, but renal involvement can occur, although very rare, causing a spectrum of symptomatology [4, 5]. Renal echinococcosis is primarily treated surgically [5], but current practices lack a solid consensus on other treatment modalities for patients who opt out of surgery, and data on their effectiveness are largely debatable and scarce. In this report, we are presenting a peculiar case of renal echinococcosis presenting with gross hydatiduria and extensive parenchymal destruction treated with exclusive dual antiparasitic therapy after refusing surgery. We present this case for its rarity and the challenges associated with its atypical treatment approach. We aspire through this report to fill significant gaps in the therapeutic management of renal echinococcosis and guide the establishment of reliable recommendations regarding its pharmacological treatment, especially when surgical options are unfeasible.

## Case presentation

**Fig. 1:**
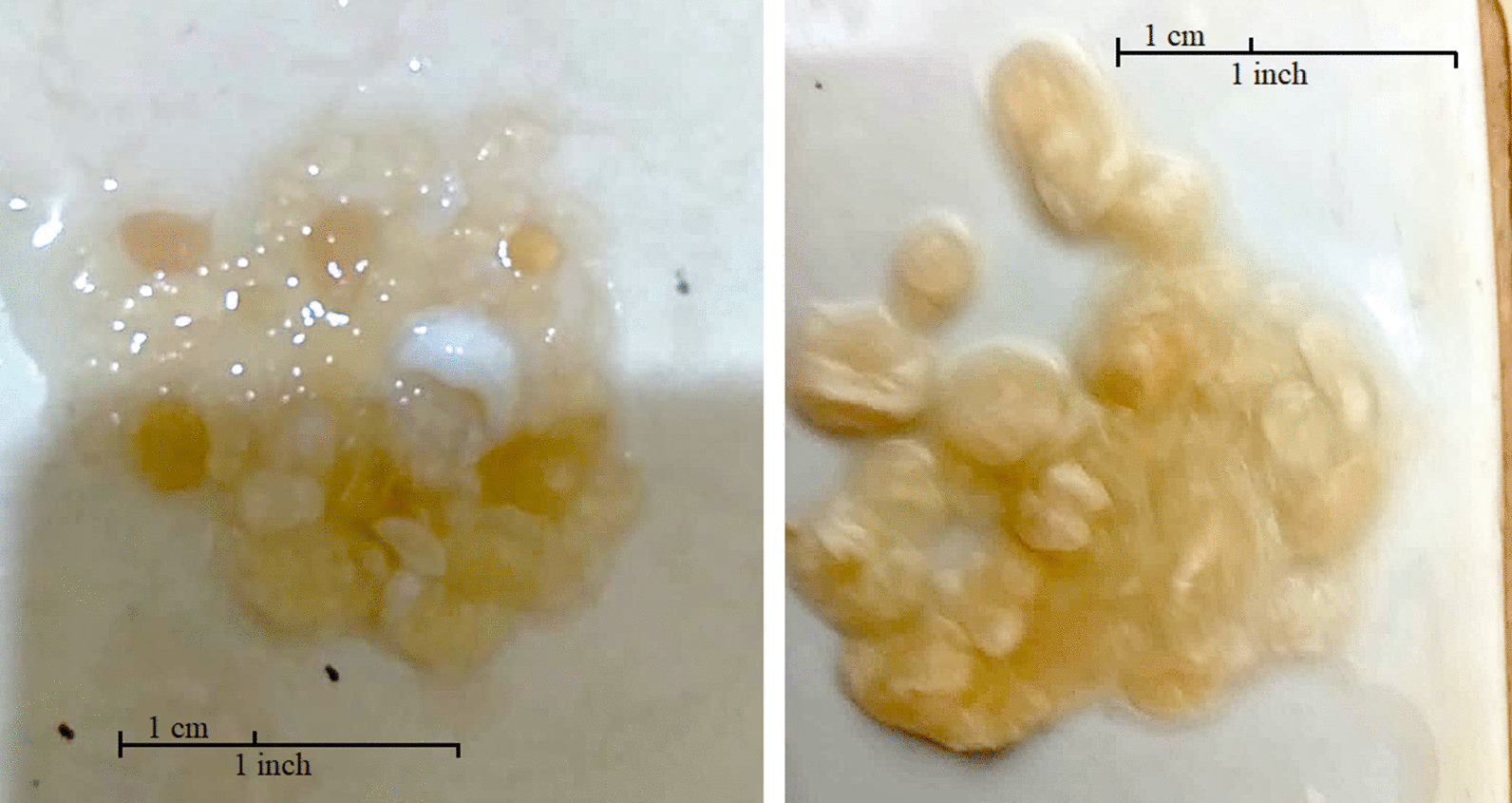
Gross hydatiduria as evidenced by passing cream-colored grape-skin-like structures in urine representative of daughter cysts

A 49-year-old Syrian male patient presented to the outpatient infectious diseases clinic at our hospital with a chief complaint of acute right flank pain associated with dark urine as well as passage of several stones and cream-colored grape-skin-like structures in urine (Fig. 1). No hematuria or other lower urinary tract symptoms were present. Of importance, the patient reported a remote history of hepatic hydatid cyst several years ago that was managed with percutaneous aspiration–injection–reaspiration (PAIR) drainage and albendazole that he sought to discontinue after several months of use. He acknowledged failing to seek medical follow-up owing to financial constraints after symptom resolution and no further recurrence. The patient is a shepherd by profession, spending most of his adult life in Syria before fleeing the civil war in 2015 and immigrating to Kuwait. He has a long-standing history of tobacco dependence, but the rest of the history is otherwise unremarkable.

At the time of his initial visit, the patient did not appear to be in acute distress or in pain. He was afebrile and hemodynamically stable with a blood pressure of 134/78 mmHg and a heart rate of 76 beats per minute. Abdominal examination was remarkable for right flank tenderness to percussion with no evidence of hepatosplenomegaly. No abdominal masses or signs of peritoneal irritation were noted. The rest of the examination was normal. The patient was admitted for a workup and further management.

**Fig. 2:**
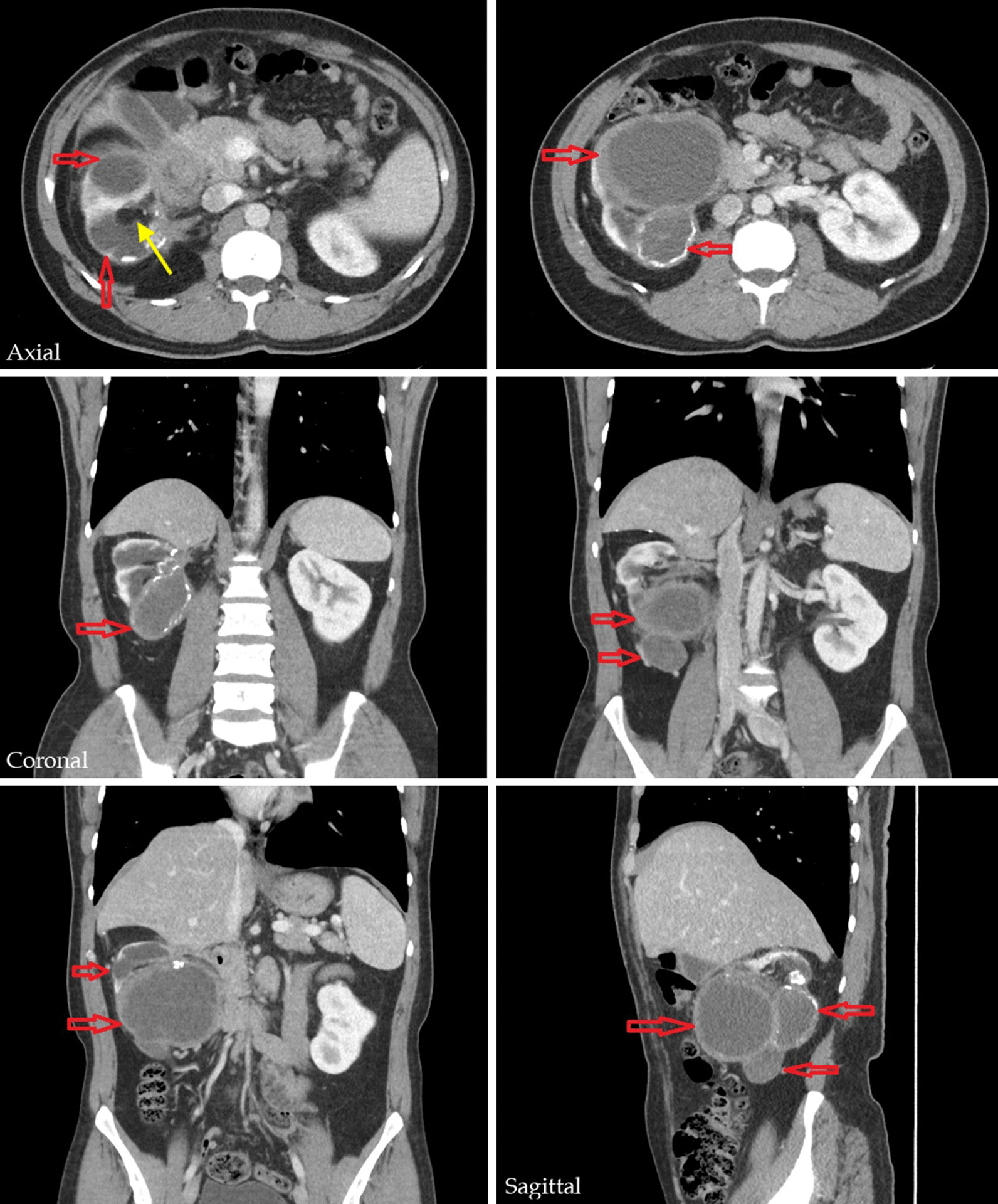
Contrast-enhanced CT showing three hydatid cysts occupying most of the parenchyma of the right kidney (red arrows) with evidence of cyst communication with the pelvicalyceal system (yellow arrow)

Initial laboratory workup showed no evidence of leukocytosis or eosinophilia. His serum creatinine was 1.03 g/dL (normal value: 0.9–1.3 g/dL) and hemoglobin was 13.6 g/dL (normal value: 14–16 g/dL). Electrolytes were normal and so were liver function tests. Urinalysis showed excess of leukocytes and microscopic hematuria (6–7 red blood cells/high-power field), with no bacterial growth on culture. Calculated glomerular filtration rate (GFR) was 81 mL/minute/1.73 cm^2^ (normal value >90). Excreted stones were collected and sent for analysis, which later showed a mixed composition of calcium oxalate and urate. Hydatid serology using *Echinococcus* indirect hemagglutination assay (IHA) (ELI.H.A, kit number 66604) was run once and came back positive at a titer of 1:2048. Imaging was obtained next. Kidney, ureter, and bladder (KUB) x-ray was normal while a renal ultrasound showed multiple unilocular hypoechoic cystic structures in the right kidney with mixed homogeneous and heterogeneous components. Contrast-enhanced computed tomography (CT) KUB revealed three sizeable thick-walled exophytic cystic lesions occupying the mid and lower zones of the right kidney with heterogeneous, nonenhancing, and hyperdense components (Bosniak category III), with the largest measuring 9 × 8 × 7.5 cm and compressing the pancreatic head, right colon, and second part of the duodenum (Fig. 2). Occasional border calcification and communication between the cyst and the pelvicalyceal system (yellow arrow) were also seen.

Of concern, the cystic lesions were encroaching the right renal hilum, resulting in renal pelvic compression and grade III hydronephrosis. The calyces were filled with debris and a small nonobstructing stone measuring 11 × 4 mm. By contrast, the left kidney was entirely normal and pathologically uninvolved. Consistent with his previous hepatic involvement, a defective segment of the right hepatic lobe with border calcification was also seen and is suggestive of prior intervention to treat the hepatic cyst. In light of the CT scan findings, referral to the urology service was initiated, and the patient subsequently underwent dynamic renal scintigraphy to assess the function of the right kidney. Scintigraphy revealed a markedly decreased tracer uptake and poor excretion indicative of negligible function (5%) of the right kidney due to extensive parenchymal destruction and replacement by the cysts as opposed to the well-functioning left kidney. The CT and scintigraphy findings strongly suggest nonsalvageability of the right kidney but without evidence of overt renal failure as supported by the normal serum creatinine and only slightly decreased GFR due to noninvolvement of the left kidney.

Based on the patient’s suggestive occupational history, past history of hydatid disease, positive serology, pathognomonic presentation of hydatiduria, and CT KUB findings, a preliminary diagnosis of renal echinococcosis with significant unilateral renal impairment was made. A diagnosis of necrotic renal cell carcinoma was briefly considered given the heterogeneous nature of the lesion, presence of calcification, and the patient’s smoking history, but the constellation of the above-mentioned findings renders a malignancy diagnosis less likely. Of note, border calcification can be detected in hydatid cyst and, when complete, is indicative of quiescence or death of the parasite [6]. Inherited cystic disorders of the kidney, such as polycystic kidney disease, tuberous sclerosis, and Von Hippel–Lindau syndrome, were unlikely due to unilateral involvement, sparing of other organs, and lack of positive family history. The possibility of multiple renal abscesses was raised, but the patient’s unsuggestive symptoms, marginally abnormal urinalysis, and normal urine culture moved it lower on the list.

After collecting data from the patient’s history and investigations and consulting with the urology service, the patient was thoroughly informed of his diagnosis, prognosis, and available surgical treatment options. Due to kidney nonsalvageability, unilateral nephrectomy was recommended to allow for complete eradication of the infection and prevention of recurrence; he was elaborately informed of the risks, benefits, and alternatives to surgical intervention, including PAIR and exclusive medical therapy. The patient chose to opt out of surgery and settle for exclusive pharmacotherapy. Reasons for surgery refusal are reported in the patient perspective section below. The patient was started on dual antiparasitic therapy with albendazole (400 mg × 2 day) and praziquantel (40 mg/kg once weekly). He was also advised to increase fluid intake and take acetaminophen on an as-needed basis for pain.

The patient decided to continue seeking follow-up medical care in his home country and probably consider undergoing PAIR in Syria. From our standpoint, the plan is to continue treatment with albendazole and praziquantel for 3–6 months duration for now and repeat serology titers and imaging to evaluate disease progression and response to treatment. After initiating therapy, the patient continued to follow up at our clinic for 4 months and reported complete resolution of symptoms, including hydatiduria. Follow-up investigations, including serum creatinine, remained normal whereas repeated *Echinococcus* serology showed a dramatic decline in titers by 50% to 1:1024, which collectively indicate clinical and serologic improvement and a satisfying response to therapy till this point. The patient confirmed his adherence to medications and reported no adverse effects and was therefore asked to continue his pharmacotherapy. The patient refused to undergo additional imaging to assess for radiological improvement in the meantime but has expressed his will to keep the team informed of his follow-up updates in his home country.

## Discussion and conclusions

Human echinococcosis is a parasitic infection caused by the larvae of the *Echinococcus* cestode. While this genus contains various species, chief among them are *E. granulosus* and *E. multilocularis*, causing cystic and alveolar echinococcosis, respectively. *E. granulosus* has a rather complex life cycle that involves a definitive host, typically a dog, and an intermediate host, which can accidentally be a human. Individuals in certain endemic regions are at risk, particularly shepherds who come in close contact with sheepdogs. Upon fecal–oral transmission of ova, larvae hatching in the small intestine enter the circulation and typically migrate to the liver and lungs where they remain dormant in encysted forms (primary echinococcosis). If a hydatid cyst ruptures, liberated protoscolices can disseminate to various organs where they establish a secondary disease or manifest acutely with rupture symptoms that range from fever and urticaria to a full-fledged anaphylactic shock [4].

**Fig. 3:**
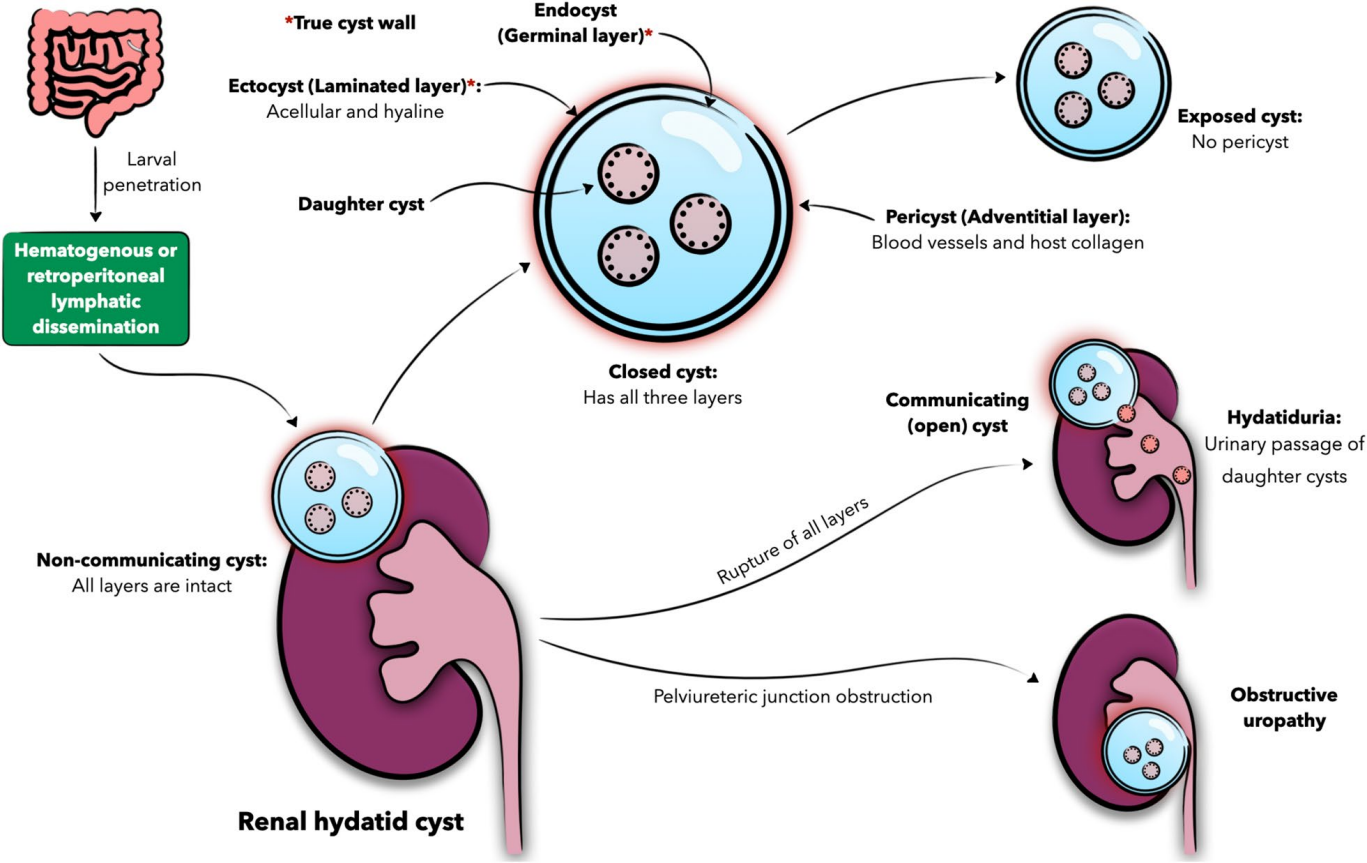
Schematic presentation of the pathophysiology, pathologic anatomy, and possible manifestations of renal echinococcosis. The figure was created by AA, an author of this manuscript

Renal involvement of *E. granulosus* infection is quite rare with an incidence as low as 2–3% of patients with cystic echinococcosis [5]. If documented, it often occurs in the setting of disseminated disease through hematogenous or retroperitoneal lymphatic spread alongside more commonly involved organs, but isolated renal cystic echinococcosis, although even rarer (1.9%), has been reported in the literature [7]. A renal hydatid cyst is typically solitary in the upper or lower poles of the kidney and cortical in location. Although often asymptomatic, patients with particularly large renal cysts (> 10 cm) can manifest a spectrum of signs and symptoms that range from flank discomfort, palpable mass, and hematuria to acute renal colic, hypertension, and even renal failure [8–10]. Hydatiduria, or the passage of daughter cysts in urine, is an uncommon (10–20% of cases) yet pathognomonic feature that follows cyst rupture into the pelvicalyceal system (Fig. 3) and has been the presenting complaint of our patient [5]. Although often microscopic, our patient had gross hydatiduria, increasing the diagnostic suspicion of renal involvement and lending support to imaging findings [9]. Renal hydatid cyst can be either closed or exposed based on layer structure, and noncommunicating or open based on layer integrity as shown in Fig. 3 [11].

Laboratory tests revealing eosinophilia (in only 50% of cases) and a positive serology are helpful in narrowing down the list of differentials, especially with a relevant exposure history, but imaging is fundamental for establishing a semi-definitive diagnosis of renal disease, especially when serology is negative [5]. A renal ultrasound may reveal the presence of cortical cysts, daughter cysts, floating membranes, and hydatid sand (Gharbi classification), but a CT scan, with a sensitivity as high as 98%, is far more yielding and helps assess for anatomic integrity and extrarenal disease [12]. Interestingly, our patient had concurrent urinary passage of stones, resembling a previous case report with similar findings [9] and two other reports of an incidental renal hydatid disease in patients treated for nephrolithiasis [13, 14]. While this may be due to chance alone, it would be intriguing to learn whether renal hydatid disease fosters stone formation in the urinary tract and to attempt to elucidate the mechanistic basis of this association, if any, that may or may not be similar to other stone-promoting infections. Another possibility is that the encroaching cysts may cause obstructive uropathy and urinary stasis that favor nidus formation and stone precipitation.

Although surgery is the cornerstone of renal echinococcosis treatment, many factors come into play when choosing among surgical options or, rather, sufficing with medical treatment, including cyst location and size, degree of parenchymal destruction, and, of course, patient’s preference [5]. Surgical options include simple nephrectomy and partial nephrectomy, which can be performed laparoscopically with impressive postoperative outcomes [15, 16]. Nevertheless, kidney-sparing surgery, such as cystectomy or pericystectomy, should be sought whenever feasible over radical surgery to preserve the renal function [17]. If the cyst extends into the pelvicalyceal system, endocystectomy with defect closure may also be performed [5]. Perioperative albendazole should also be started as a therapeutic adjunct to decrease recurrence and prevent anaphylaxis, the risk of which could also be minimized by administering intraoperative scolicidal agents [17]. One month of preoperative albendazole diminishes larval activity and is continued for three more months postoperatively to decrease recurrence [18]. If surgery is conducted laparoscopically, a retroperitoneal approach could decrease peritoneal seeding if the cyst ruptures accidentally, but as malignancy cannot be excluded with surety preoperatively, a transperitoneal approach may be a preferred option [17]. PAIR of a renal hydatid cyst is a less commonly employed modality that has been reported in the literature with a debatable success rate and high risk of peritoneal seeding and anaphylaxis that may limit its use to surgically unfit patients [19].

Although extensive parenchymal destruction and cyst extension, as evidenced by hydatiduria, necessitate surgical intervention, our patient did not opt for surgery and sought to receive antiparasitic therapy alone. Exclusive medical therapy for renal hydatid disease is considered a rather uncommon practice. In fact, solid guidelines that delineate accurate indications and regimens of pharmacotherapy for renal echinococcosis do not currently exist. Case reports describing exclusive medical therapy for renal hydatid disease are otherwise sparse compared with its hepatic or pulmonary counterparts, with no current consensus on the circumstances under which utilization of pharmacotherapy alone would be sufficient. Furthermore, if used exclusively, dose and duration of albendazole treatment should be tailored to the medically treated patient with renal echinococcosis to ensure higher eradication rates that would otherwise be achieved more conveniently with surgery.

In a previous retrospective study done to investigate the incidence of unusual locations of hydatid disease, it was reported that a female patient with a symptomatic small renal hydatid cyst has received pharmacological treatment alone and improved [20]. Although promising, the clinical rationale behind resorting to exclusive medical therapy has not been elucidated, neither have been the parameters for assessing improvement in this patient. A case report described a 14-year-old patient with renal hydatid disease who was exclusively treated with four 4-week cycles of albendazole, which resulted in seronegativity and marked reduction in cyst size (from 9 × 9 × 6.8 cm to 3.6 × 3.4 × 2.3 cm) [21]. On the other hand, another report is of a 79-year-old patient with a renal hydatid cyst who refused surgery and was started on daily praziquantel and albendazole for 26 months before becoming seronegative and was therefore asked to stop the medications. Aside from a single recurrence, the patient showed significant improvement in cyst size and symptoms using exclusive dual pharmacotherapy [12]. Whereas albendazole halts cyst growth, praziquantel kills gastrointestinal worms and larval protoscolices, preventing the formation of secondary cysts [22, 23]. A meta-analysis evaluating different treatment approaches of cystic echinococcosis indeed showed higher scolicidal and anticystic activity, and therefore improvement and cure rates, when both medications are used in combination compared with albendazole alone. In addition, dual therapy may reduce the risk of recurrence and intraperitoneal seeding if rupture or spillage occurs [23]. In another nonrandomized quasi-experimental study, patients with multiple hydatid cysts were treated with three 4-week cycles of albendazole and praziquantel and were followed up for an average of 18 months after treatment. Clinically, symptoms resolved in 77.8% of patients and partially so in 22.2%. Radiologically, significant improvement was noted in 55.6% while partial improvement was seen in 44.4% [24]. However, the study sample was not sufficiently representative of patients with renal disease.

Despite the overall promising outcomes, unpredictable responses and adverse effects of medical therapy may render its use for renal hydatid disease less preferable than surgery but may still be considered in younger patients to spare the kidneys, in disseminated disease, and in those who either opt out of or are not fit for surgical intervention [21]. As the risk of recurrence is higher with exclusive pharmacotherapy compared with surgery, treatment may continue for extended durations that are tailored based on disease progression and response to treatment [25]. Further randomized controlled trials are therefore strongly warranted to examine the efficacy and indications of exclusive combination therapy for renal echinococcosis, appropriate dosing regimens, and sufficient duration of treatment (for example, 3–6 months versus 12–24 months versus lifelong.) Not only would this delineate cost-effective and least harmful regimens but also help establish reliable guidelines and treatment recommendations that will ground future clinical decisions that we may now find dilemmatic. While a limitation of this report may be the relatively short follow-up period, exclusive medical therapy has, nevertheless, proved effective when our patient was assessed clinically and serologically.

Several preventive measures could also be taken to prevent transmission and acquisition of echinococcal infection, particularly in high-risk groups. The centers for disease control and prevention have laid out few recommendations in this regard that include preventing dogs from feeding on the carcasses of infected sheep, controlling stray dog populations, and restricting home slaughter of sheep and other livestock [26]. In addition, avoiding consumption of food or water that may have been contaminated by dog fecal matter, washing hands after handling dogs, and teaching children the importance of hand hygiene may have substantial benefits in preventing infections in the long run [26]. An additional preventive approach is treating dogs with anthelmintics, namely praziquantel, which help in breaking the parasite life cycle and reducing human exposure [27]. Although vaccination of dogs, the definitive hosts, has been evaluated as a preventive measure by several studies, there is little evidence on the efficacy of this approach, with most reports yielding inconsistent results [27]. However, it seems that vaccination of intermediate hosts such as sheep may be a reliable and effective strategy. In fact, it is suggested that a combination of vaccination with EG95, an oncosphere antigen, with 6-monthly treatment of dogs with praziquantel may achieve timely and robust control of cystic echinococcosis transmission [27].

This case is a prime example of the challenges that physicians face in practice when a trade-off between standard treatment approaches and patient’s autonomy needs to be achieved. In our case, the patient declined nephrectomy, which he perceived to be more burdensome than beneficial and was therefore offered medical treatment. Although patients’ autonomous decisions may not always be in their best interest, they should still be thoroughly counseled and offered other reasonable options after weighing the scientific evidence. While data on exclusive medical therapy for renal echinococcosis are largely lacking, our report indicates promising early outcomes of dual antiparasitic pharmacotherapy with albendazole and praziquantel, potentially guiding future trials aimed at outlining reliable recommendations for patients treated with this approach.

### Patient perspective

“I am not surprised of having this diagnosis. I have lived with it for decades and come to accept living with these cysts. God will take care of me; I trust God with this and depend on you, my doctors. In fact, I feel like I am improving. The cysts I have been passing in urine have decreased and I am no longer passing stones. I am not in pain anymore either. Everything is going well. However, I do not accept or believe the results of the renogram. There is no way that my right kidney is functioning at 5%. As soon as it is safe to travel, I intend to go back home, to Syria. I will visit my old urologist to have him perform PAIR of my kidney cyst. I believe PAIR cured my liver cyst years ago, and it will cure me again. In the meantime, I intend to continue albendazole and praziquantel that you prescribed me. I think they are working perfectly to relieve my symptoms. This whole experience has been stressful, but I have faith in God and that everything was meant to happen and for a reason. God will protect me.

The financial aspects, however, were the hardest part. The medications are very expensive, and I could barely afford them. Thank you for helping me out; I do not know what I could have done without you. Regarding the surgery, there is no way I am going to agree to undergo a nephrectomy. I believe my condition will be treated with albendazole and praziquantel, and maybe PAIR if I seek it later. Nephrectomy will be drastic for me and I will never accept it. My cousin underwent a nephrectomy and he is now dialysis dependent. No way I will end up like him!” When the patient was asked to sign the consent form, he added: “I am so glad others will learn from my condition. This will create hope and facilitate easier treatments for other patients with a similar diagnosis. However, I have one request; do not share images of my renogram in your article, please. I do not want others suffering from the same disease to freak out; I want my condition to give them hope, and I do not want them to think that nephrectomy is the only option.” Abiding by the patient’s request, images of his renal scintigraphy were not included in this report.

## Data Availability

All relevant data and materials of our patient’s case are included in this published article.

## Abbreviations

WHO: World Health Organization
PAIR: Percutaneous aspiration–injection–reaspiration
GFR: Glomerular filtration rate
IHA: Indirect hemagglutination assay
KUB: Kidney, ureter, and bladder
